# Antipsychotics dosage and antiparkinsonian prescriptions

**DOI:** 10.1186/1745-0179-3-14

**Published:** 2007-09-03

**Authors:** Eric Acquaviva, Isabelle Gasquet, Bruno Falissard

**Affiliations:** 1 INSERM U669, PSIGIAM (Paris Sud Innovation Group in Adolescent Mental Health Methodology), 97 boulevard de Port-Royal, 75679 Paris cedex 14, France; 2 Direction de la Politique médicale, Assistance Publique – Hôpitaux de Paris, 3 avenue Victoria, Paris, France

## Abstract

**Background:**

To study the link between the dosage of several antipsychotics and the prescription of antiparkinsonians in an observational study.

**Methods:**

In the context of a national naturalistic prospective observational study, a database containing all the prescriptions from 100 French psychiatrists during the year 2002 was analysed. The inclusion criteria were a diagnosis of schizophrenia or schizoaffective disorder and age over 18. The mean dosage of antipsychotics with and without antiparkinsonians was compared. Since there were multiple prescriptions for a given subject, generalised mixed linear models were also used to study the link between antiparkinsonian prescription and antipsychotic dosage.

**Results:**

antiparkinsonians were prescribed to 32,9% of the patients. Two groups of antipsychotics were observed relating to differences in dosage when an antiparkinsonian was co prescribed or not : a first group, where the mean dosage was higher with antiparkinsonians (risperidone, amisulpride and haloperidol) and a second group (clozapine, olanzapine), in which antiparkinsonian co prescription was not related to the dosage of antipsychotics.

**Conclusion:**

As a conclusion, it can be said that it is important to consider the dosage and the type of antipsychotic in the treatment of patients suffering of schizophrenia, because neurological side effects are frequent and can impair quality of life. Moreover the prescription of antiparkinsonians can lead to different side effects such anticholinergic effects.

## Background

Over the last decade, a new generation of antipsychotics has emerged. In certain randomised trials, these new antipsychotics has been proved to cause less neurological side effects than traditional antipsychotics[1-4]. Nevertheless, recent meta-analyse tend to show that only certain classes of atypical antipsychotics (olanzapine and clozapine) lead to fewer adverse neurological events[5-7]. These meta-analyses point out that the dosage of antipsychotics is decisive for the occure of adverse neurological events. Because randomised trials study antipsychotics for one or several different dosages, these studies have limitations in the analysis of the link between neurological adverse events and antipsychotic dosage [1].

Observational studies make it possible to study antipsychotic dosage as it is prescribed in ordinary practice. In addition, antiparkinsonians are prescribed for neurological side effects in schizophrenia and the frequency of their prescription reflects the frequency of neurological side effects[8].

The aim of this analysis is to study the link between antipsychotic dosage and the prescription of antiparkinsonians in an observational study.

## Method

### Data sources

Data for the study is derived from the Thales company database. This company has developed an exhaustive computerized medical file system used by a panel of randomised French psychiatrists. For each consultation, treatment and diagnosis were entered directly by the psychiatrist. Diagnoses were made in the context of day to day practice, without the use of standardized interviews. The software provided printouts of prescriptions and recorded data in a central data base. The practitioners were not paid for their participation.

### Sample

The psychiatrists in the panel were private practitioners. They were chosen at random with stratification for area of practice, gender and age. Other characteristics like salary, the kind of practice (private only, private and public practice) were compared with the French national social security register. Regarding all these characteristics, the panel of 100 psychiatrists was representative of the French population of private practitioners.

All the subjects included were outpatients. Inclusion criteria were as follow: patients over 18 who consulted any psychiatrist on the panel at least once in the period from 02/01/01 to 02/12/31 and who had at least once received a diagnosis of schizophrenia or schizoaffective disorder, on the DSM-IV criteria[9].

The variables retained in our analysis were socio-demographic data (age, gender), diagnosis (schizophrenia, schizoaffective disorder, other associated diagnosis) and medication. Any medications of potential interests for the object of study was retained (olanzapine, risperidone, clozapine, amisulpride, haloperidol).

Olanzapine, risperidone, clozapine, amisulpride were considered as atypical antipsychotics whereas haloperidol was considered as a typical antipsychotic.

### Analysis

In a first step, the frequency of prescription was determined for each antipsychotic and antiparkinsonian. The mean dosage of antipsychotics with and without antiparkinsonians was also calculated.

In a second step, the frequency of antiparkinsonian co prescriptions for different dosages of several different antipsychotics, converted into chlorpromazine dosage equivalent, was studied.

Since there were multiple prescriptions for given subject, generalised linear mixed models were also used. Furthermore, different antipsychotics could be co prescribed. Thus, the dosage of antipsychotics studied as variables of interest and the co prescription of antipsychotics other than haloperidol, olanzapine, risperidone, amisulpride and clozapine were entered into the model as confounding variables, as well as age and gender. The prescription of antiparkinsonians was considered as the dependant variable [10-13].

For statistical analyses, SAS 8.2 software was used, proc gen mod was used for the mixed models[14].

## Results

During 2002, 2523 patients consulted a psychiatrist from the panel of 100 psychiatrists at least once. 922 subjects suffering from schizophrenia or shizo-affective disorder were included. 85,6% received a diagnosis of schizophrenia at least once in 2002, 31,9% a diagnosis of schizo-affective disorder, and 11,8% an associated diagnosis of affective disorders. 28,9% of patients had at least two different diagnoses. All subjects included were prescribed medication at least once.

### Demographic characteristics

There were no missing data for the variable gender and 2 missing data for age. Mean age was 39.7 (min = 18, max = 90, sd = 12.0). Men were younger than women (38,2 vs 41,8 ; p < 0,01) and represented 57.9% of the sample population.

In order to check the representative ness of the sample of patients, demographic data were compared with referential data from another French naturalistic study [15]. There was no significant difference considering the sex ratio (57,9% vs 60,9% ; p = 0,1) and no significant difference between mean age (39,7 vs 39,2; p = 0,2).

### Prescriptions

On average, a patient received 6.0 prescriptions in the year 2002 (sd = 2,2). The mean number of psychotropic medications per prescription was 2.6 (sd = 1,1). Antiparkinsonians were prescribed to about one third of the patients (table 1).

**Table 1:** Frequency of antipsychotic and antiparkinsonian prescriptions

	% of patients who have received the treatment at least once in 2002 (n = 922)		% of prescriptions where the treatement is prescribed (n = 5712)	
Treatment	%	n	%	n
olanzapine	28.6	264	22.4	1278
risperidone	19.1	176	12.5	713
amisulpride	20.5	189	16.9	967
clozapine	0.8	7	0.8	44
haloperidol*	16.6	153	8.1	462
antiparkinsonians	32.9	303	27.3	1564

* oral or depot

13,6% of olanzapine prescriptions are associated with antiparkinsonians. Haloperidol is more often co prescribed with antiparkinsonians (38,2% of the prescriptions of haloperidol) than atypical antipsychotics (17,3% for risperidone, 35,2% for amisulpride). For clozapine, only three cases of antiparkinsonian co prescriptions are recorded.

Table 2 indicates the characteristics of dosage for the different antipsychotics.

**Table 2:** Mean dosage of antipsychotics according to co-prescription of antiparkinsonians (univariate analysis)

		All prescriptions (n = 5712)		Prescriptions with no antiparkinsonian (n = 4148)		Prescriptions with antiparkinsonian (n = 1564)		Comparisons of means	
	*n*	Mean dosage mg (chlorpromazine equivalents) [CI95%]	Range of dosage Median (min-max)	*n*	Mean dosage	*n*	Mean dosage	t	p
	
olanzapine	*1278*	10,9 (218) [10,6–11,3]	10 (5–30)	*1104*	*10,7*	*174*	*11,3 *	*0,84*	*0,4*
risperidone	*713*	3,5 (175) [3,3–3,7]	4 (0,5–12)	*590*	*3,2*	*123*	*5,3 *	*7,8*	*<0,0001*
amisulpride	*967*	365 (243) [346–382]	200 (50–1200)	*627*	*298*	*340*	*468,2 *	*10,1*	*<0,0001*
clozapine	*44*	90,6 (181) [75,8–115,5]	75 (12,5–450)	*41*	*89,9*	*3*	*100,0 *	*0,8*	*0,05 *
haloperidol	*452*	6,6 (330) [6,1–7,2]	5 (0,1–30)	*173*	*3,9*	*279*	*8,4 *	*68,9*	*<0,0001*

### Comparison of dosage between antipsychotics prescribed with and without antiparkinsonians (table 3)

**Table 3:** Generalised linear mixed models

	β*	p
Olanzapine dosage	0,0011	0,64
Risperidone dosage	0,01	<0,0001
Amisulpride dosage	0,0035	0,005
Clozapine dosage	0,0007	0,67
Haloperidol dosage	0,01	0,009
Multiple prescriptions of antipsychotics	0,0009	0,37
Age of the patient	0,0008	0,23
Gender of the patient	0,003	0,15

* Represents the coefficient of the link between the prescription of antiparkinonians (the dependant variable) and the dosage of the antipsychotic or age, gender or the co prescription of another antipsychotic.

Two groups of antipsychotics were observed: a first group (risperidone, amisulpride and haloperidol) where mean dosage was higher with antiparkinsonians, and a second group, in which the dosage of antipsychotics (clozapine, olanzapine) did not differ with or without antiparkinsonians (table 3).

### Percentage of antiparkinsonian co prescription in relation to antipsychotic dosage

**Figure 1:**
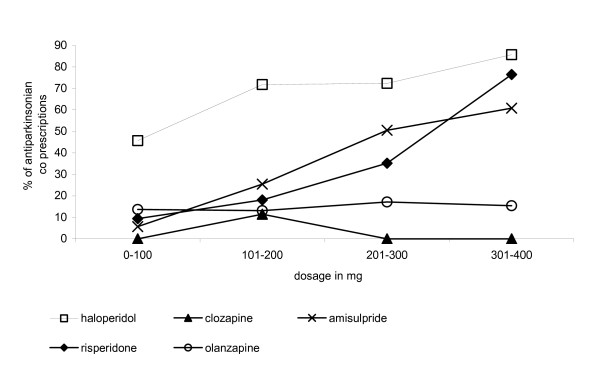
Antipsychotics dosage and antiparkinsonian prescriptions. 100 mg equivalent dosage in chlorpromazine = 10 mg of olanzapine. = 50 mg of clozapine. = 5 mg of haloperidol. = 3 mg of risperidone.

Figure 1 indicates the evolution of the percentage of antiparkinsonian co prescriptions in relation to antipsychotic dosage. A stable percentage of antiparkinsonian co prescriptions for the different doses of olanzapine was observed. Concerning risperidone and amisulpride, the increase in antiparkinsonian co prescriptions is proportional to the dosage of risperidone.

### Factor influencing antiparkinsonian prescription (generalized mixed linear models)

The other antipsychotics co prescribed with each of antipsychotics studied (olanzapine, risperidone, clozapine, amisulpride and haloperidol) did not appear to be significant confounding variables.

The results of the comparison of means are confirmed. Antiparkinsonian co prescriptions increase with the dosage for amisulpride, risperidone and haloperidol whereas they are independent of the dosage for olanzapine and clozapine.

## Discussion

This naturalistic study is likely to be more representative of day to day practice than randomised trials, in terms of selection of population, number of prescriptions studied, dosage and time of follow up[8]. This kind of study has nevertheless certain limitations, among which the lack of randomisation.

Despite the emergence of a new generation of antipsychotics, the co-prescription of antiparkinsonians is still high (32,9%). This probably means that neurological side effects are a real problem for about one third of the patients treated with antipsychotics.

This analysis underlines the importance of the dosage of antipsychotics for the occurrence of neurological side effects. The patients under higher doses of amisulpride or risperidone (over 300 mg in chlorpromazine dose equivalent) are as likely to need antiparkinsonians as those under a traditional neuroleptic as haloperidol. Considering dosage of olanzapine and clozapine, the occurence of neurological side effects seems to be stable.

Another important point is that it is known that the combination of several antipsychotics increase the frequency of antiparkinsonian prescription [18-20] but the presence of another antipsychotic does not appear decisive in this study for the link between dosage of antipsychotics and prescription of antiparkinsonians.

As a conclusion, it can be said that the difference between atypical and typical antipsychotics is confirmed here in terms of the percentage of antiparkinsonian co prescriptions and hence neurological side effects. However, these differences depend on the dosage of antipsychotics.

This point is important in considering the treatment of patients suffering of schizophrenia because neurological side effects impair quality of life. It is also important because the prescription of antiparkinsonians can lead to different adverse effects such anticholinergic effects.

## Authors' contributions

BF and EA conceived of the study and participated in its design and coordination. IG and EA participated in the design of the study. EA performed statistical analysis. All authors read and approved the final manuscript
